# Erianin against *Staphylococcus aureus* Infection via Inhibiting Sortase A

**DOI:** 10.3390/toxins10100385

**Published:** 2018-09-23

**Authors:** Ping Ouyang, Xuewen He, Zhong-Wei Yuan, Zhong-Qiong Yin, Hualin Fu, Juchun Lin, Changliang He, Xiaoxia Liang, Cheng Lv, Gang Shu, Zhi-Xiang Yuan, Xu Song, Lixia Li, Lizi Yin

**Affiliations:** College of Veterinary Medicine, Sichuan Agriculture University, Chengdu 610000, China; ouyang.ping@live.cn (P.O.); xuewen-he@hotmail.com (X.H.); yuanzhongwei_sicau@163.com (Z.-W.Y.); yinzhongq@163.com (Z.-Q.Y.); fuhl2005@sohu.com (H.F.); juchunlin@126.com (J.L.); hecl@sicau.edu.cn (C.H.); liangxiaoxia@sicau.edu.cn (X.L.); lvcheng1980@163.com (C.L.); dyysg2005@sicau.edu.cn (G.S.); zhixiang-yuan@hotmail.com (Z.-X.Y.); songx@sicau.edu.cn (X.S.); lilixia905@163.com (L.L.)

**Keywords:** sortase A, *Staphylococcus aureus*, erianin, inhibitor, molecular mechanism

## Abstract

With continuous emergence and widespread of multidrug-resistant *Staphylococcus aureus* infections, common antibiotics have become ineffective in treating these infections in the clinical setting. Anti-virulence strategies could be novel, effective therapeutic strategies against drug-resistant bacterial infections. Sortase A (srtA), a transpeptidase in gram-positive bacteria, can anchor surface proteins that play a vital role in pathogenesis of these bacteria. SrtA is known as a potential antivirulent drug target to treat bacterial infections. In this study, we found that erianin, a natural bibenzyl compound, could inhibit the activity of srtA in vitro (half maximal inhibitory concentration—IC_50_ = 20.91 ± 2.31 μg/mL, 65.7 ± 7.2 μM) at subminimum inhibitory concentrations (minimum inhibitory concentrations—MIC = 512 μg/mL against *S. aureus*). The molecular mechanism underlying the inhibition of srtA by erianin was identified using molecular dynamics simulation: erianin binds to srtA residues Ile182, Val193, Trp194, Arg197, and Ile199, forming a stable bond via hydrophobic interactions. In addition, the activities of *S. aureus* binding to fibronectin and biofilm formation were inhibited by erianin, when co-culture with *S. aureus*. In vivo, erianin could improve the survival in mice that infected with *S. aureus* by tail vein injection. Experimental results showed that erianin is a potential novel therapeutic compound against *S. aureus* infections via affecting srtA.

## 1. Introduction

*Staphylococcus aureus* (*S. aureus*), a gram-positive bacterium, is an important opportunistic pathogen in human and animals [1]. *S. aureus* can cause a range of diseases when the host has weakened immunity. Methicillin-resistant *S. aureus* (MRSA) is a resistant strain in *S. aureus*. Antibiotics have limited or no effects on MRSA infection and contribute to increased antimicrobial resistance. MRSA is seriously threatening public health worldwide, with higher morbidity and mortality rates than non-resistant *S. aureus* strains and high therapy costs [2]. New antibiotics and new therapeutic strategies are urgently needed. In recent decade, antivirulent treatment strategy has become an immediate research focus on the treatment bacteria-medicated diseases [3,4]. 

Modern research found that virulence factors play vital roles in bacterial pathogenesis. In *S. aureus*, the virulence factors assist the bacteria to adhere the surface of host mucosal surface, destroy the red blood cells and leukocytes, and evade host’s immune defenses [1]. During the infection, *S. aureus* firstly adheres to the surface of host organ tissues via its surface proteins. *S. aureus* without surface proteins cannot adhere to the host cells [1], it soon recognized by the host’s immune system [5,6]. These extracellular associated proteins secreted by the bacteria are covalently anchored to the cell wall peptidoglycan by transpeptidase to become true surface proteins [7]. Sortase A (srtA) is one of the primary surface-anchored transpeptidases in *S. aureus* [8]. In 2000, Mazmanian et al. reported that the *S. aureus* with deleted *srtA* gene had no influence on the bacterial growth, but the mutant decreased the number of surface leucine, proline, any amino acid, threonine and glycine (LEXTG)-containing proteins and reduced the ability of the bacteria to cause renal abscesses and acute infection in mouse models [9]. In a rat endocarditis model, *S. aureus srtA* mutant showed low pathogenicity [10]. Previous studies found that *S. aureus* with *srtA* gene deletion lost its ability to bind IgG, fibronectin, and fibrinogen and had reduced survival in macrophages [9,11]. The catalytic center of srtA comprises a set of amino acids residues (His120, Cys184 and Arg197) [12]. His120 and Cys184 maintain the activity of srtA [13]. Arg197 can effectively cleavage the T-G peptide bond of LPXTG-containing proteins, which are substrates of srtA [14]. srtA is a potentially promising target for treating *S. aureus* infections.

**Figure 1:**
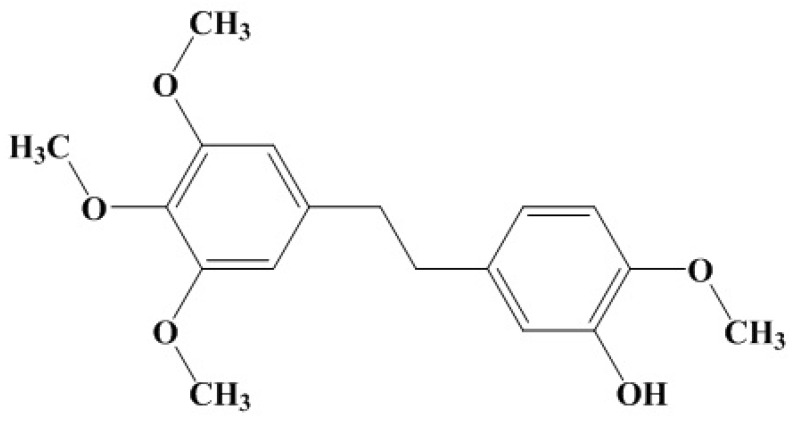
Chemical structure of erianin (CAS No. 95041-90-0).

In our group, we mainly focus on finding new compounds from traditional Chinese medicines (TCM) against *S. aureus* virulence factors. In previous researches, we found natural molecules can inhibit the α-hemolysin in vitro [15,16,17,18]. For more virulence factors, we have screened natural compounds targeting the srtA by detecting the inhibition rate of enzyme activity. We have identified several natural molecules from TCM herbs, and found that erianin had relatively high inhibitory activities. Erianin is a bibenzyl compound (Figure 1). Erianinis is also an important bioactive constituent of *Dendrobium chrysotoxum* [19]. Erianin has exhibited pharmacological antitumor by inhibiting angiogenesis [20,21,22], endothelial metabolism [23], and inflammation and by inducing the cells arrest, apoptosis, and autophagy [24,25]. Erianin has also exhibited antioxidant activity [19] and anti-benign prostatic hyperplasia [26]. On our knowledge, there are no studies focused on erianin inhibited the srtA in *S. aureus*. In this study, we evaluated the influence erianin on *S. aureus* srtA and the molecular mechanism of erianin binding to srtA. 

## 2. Results 

### 2.1. The Activity of Erianin against S. aureus SrtA

**Figure 2:**
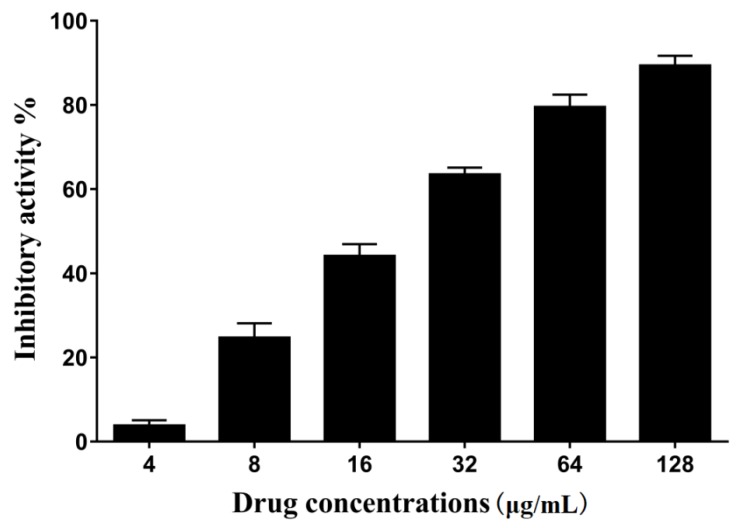
Inhibitory effects of erianin (different levels) against srtA from *Staphylococcus aureus* Newman D2C in vitro.

The fluorescent peptide Dabcyl-QALPETGEE-Edans is a substrate for srtA. Purified srtA can cleave the peptide, thus increasing the fluorescence intensity. The inhibitory activity of erianin was showed with IC_50_, which is the concentration that decreases the 50% fluorescence intensity relative to the negative control group (without erianin). The results of different concentrations of erianin against *S. aureus* srtA are presented as the percentage of inhibitory activity in Figure 2. The IC_50_ of erianin against *S. aureus* srtA was calculated to be 20.91 ± 2.31 μg/mL (65.7 ± 7.2 μM).

### 2.2. The Influence of Erianin on S. aureus Growth

**Figure 3:**
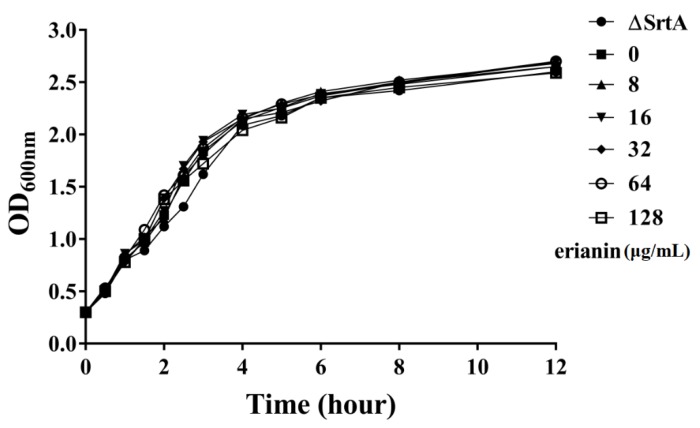
Growth curves of *Staphylococcus aureus* strain Newman D2C with or without erianin and gene knockout mutant ΔsrtA in brain-heart infusion (BHI). Symbol ●, ■, ▲, ▼, ◆, ○ and □ represent gene knockout mutant ΔsrtA, *Staphylococcus aureus* strain Newman D2C culture with 0, 4, 16, 32, 64 and 128 μg/mL erianin, respectively.

The minimum inhibitory concentrations (MICs) of erianin against *S. aureus* strain ATCC25904 (Newman D2C) and ΔsrtA strain were same (512 μg/mL). A growth curve assay was performed using strain Newman D2C with (8–64 μg/mL) or without erianin, and srtA mutant strain. The growth curves showed that erianin had no inhibitory effects on the growth of *S. aureus* at concentrations of 8–64 μg/mL (Figure 3). 

### 2.3. Erianin Reduced the Activity of S. aureus Adhesion to Fibrinogen

**Figure 4:**
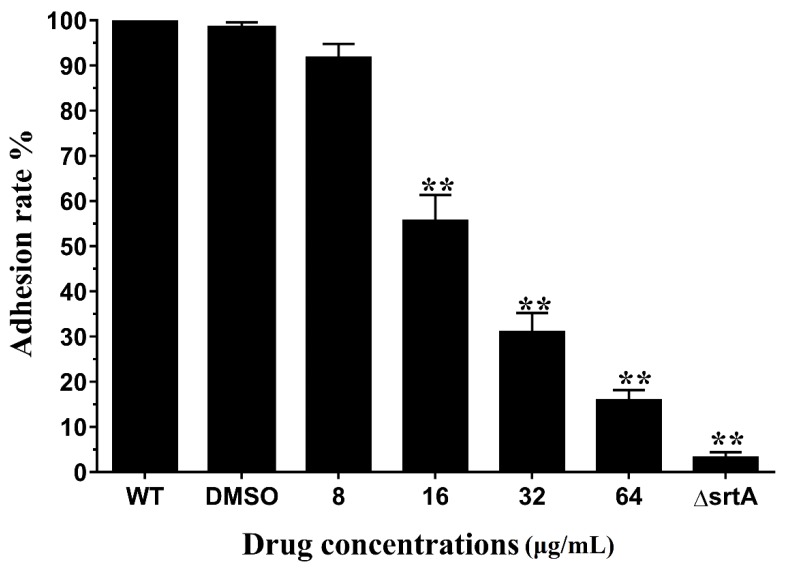
Adhesion rate of bacterial cells to Fg. erianin reduced the adhere of wild type (WT) to Fg in a dose dependent manner. Each result was derived from three independent experiments and presented as the mean ± SEM. ** *p* < 0.01 vs. the WT group.

During the early phase of *S. aureus* infection, surface proteins can adhere to host cells and invade tissues to escape the host immune defense. SrtA, a primary anchoring enzyme in gram-positive bacteria, anchors many surface proteins, such as protein A, fibronectin binding proteins, and collagen-binding proteins. Fibronectin binding proteins can invade cells via binding fibronectin/fibrinogen in host cells [27,28,29]. Erianin decrease the catalytic activity of srtA, and then surface proteins (fibronectin binding proteins) will reduce. Fg-binding assays were used to test the inhibition of binding ability of *S. aureus* srtA by erianin. The capacity of *S. aurues* Newman D2C treated with different concentrations erianin and ΔsrtA to adhere to an Fg-coat surface were examined. The results were shown in Figure 4. The adhering capacity significantly decreased in the ΔsrtA, with an Fg-adhesion rate of 3.3 ± 1.5%. The adhesion rate of *S. aurues* Newman D2C treated with 8 μg/mL erianin was lower (91.8 ± 4.1%) than that of wild type (WT) group. The Newman D2C strain treated with 64 μg/mL erianin showed a binding rate of 16.1 ± 2.9%. These results showed that erianin reduced the capacity of *S. aureus* to adhere to Fg in a dose-response manner. 

### 2.4. Erianin Decreased Biofilm Formation

**Figure 5:**
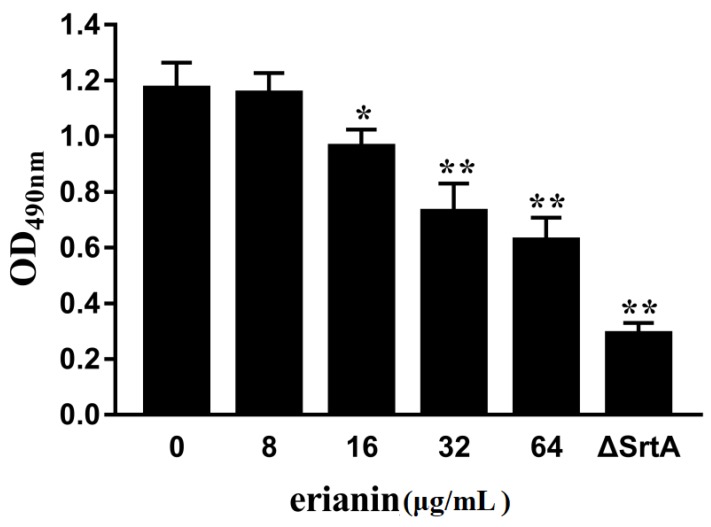
Erianin reduces the biofilm formation of *S. aureus*. Three independent experiments were performed to obtain stable results. * *p* < 0.05 vs. the WT group, ** *p* < 0.01 vs. the WT group.

The formation of bacterial biofilm is related to persistent infection in human and animals, an important factor in the failure of antibiotics [30,31]. Some surface proteins (fibronectin binding proteins, *S. aureus* surface protein C and *S. aureus* surface protein G) make a direct role in *S. aureus* biofilm formation [32]. We detected the *S. aureus* biofilm formation in the absence or presence different concentrations of erianin. *S. aureus* biofilm formations were decreased in the presence of erianin (Figure 5). When *S. aureus* treated with 64 μg/mL erianin declined significantly in the biofilm formation. Erianin affected the biofilm formation could be via inhibiting the activity of *S. aureus* srtA.

### 2.5. Mechanism Underlying the Binding of Erianin to SrtA

**Figure 6:**
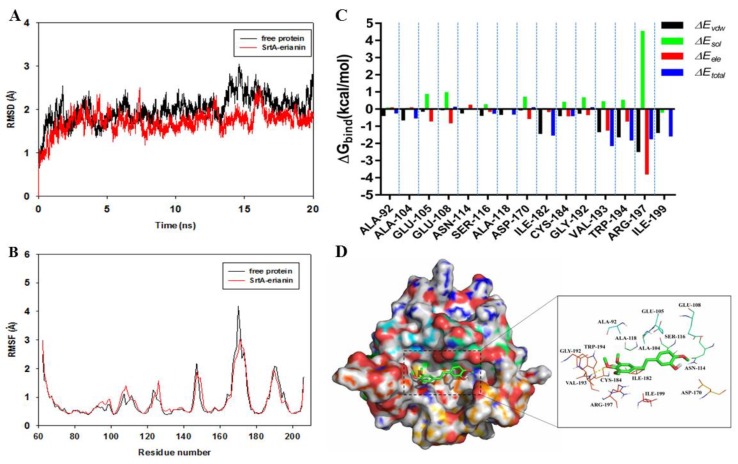
(**A**) The root-mean-square deviations (RMSDs) of all the atoms of srtA-erianin complex with respect to its initial structure as function of time; (**B**) RMSF of residues of the whole protein in srtA-erianin complex and free srtA during the 20 ns simulation; (**C**) Decomposition of the binding energy (ΔG_bind_) on a per-residue basis in the srtA-erianin complex; (**D**) The predicted binding mode of erianin in srtA binding pocket obtained from MD simulation.

Molecular docking and molecular dynamics simulations were used to explore the potential binding sites and mechanism between erianin and srtA via the AutoDock vina 1.1.2 and Amber12 software package. Based on the docking results, the mechanism of erianin binding to srtA was determined using 20-ns molecular dynamics simulations. The root mean square deviation (RMSD) values of the protein backbone were used to explore the dynamic stability of the models and ensure the rationality of the sampling strategy. RMSD values of the protein backbone were calculated based on the starting structure along the simulation time and plotted as shown in Figure 6A. We found that the protein structure of srtA and its binding with erianin were stabilized during the simulation. 

To reveal the flexibility of the residues of the whole protein in the srtA-erianin complex and free srtA, the root mean square fluctuations (RMSF) of the residues were calculated. Different flexibilities of all residues in the srtA protein with or without of erianin were shown in Figure 6B. The fluctuation patterns of the srtA-erianin complex and free srtA were different during the final 20 ns of the simulation. All of the residues in the binding site of srtA-erianin complex showed a certain degree of flexibility compared with free srtA, with RMSF of <3 Å. These results indicated that the residues in the srtA-erianin complex were more rigid.

### 2.6. Identification of the Site of Erianin Binding to SrtA 

The Molecular Mechanics Generalized Born Surface Area (MMGBSA) method was used to calculate the binding free energy of the residues surrounding the binding site to explain their contribution to the entire binding system. The binding free energies of each residue included Van der Waals (∆Evdw), solvation (∆Esol), electrostatic (∆Eele) and total contributions (∆Etotal) (Figure 6C). The Arg197 residue of the srtA-erianin complex has a strong electrostatic contribution, with the ∆Eele of <−3.0 kcal/mol. There was cation-π interaction between srtA and erianin, because Arg197 is close to the phenyl group of erianin, and electrostatic interactions exist in this complex (Figure 6D). In addition, residue Trp194, with the ∆Evdw of <−1.5 kcal/mol, has an appreciable Van der Waals interactions with erianin, leading to formation of a hydrogen bond between srtA and erianin. The Van der Waals interactions were a primary source of the decomposed energy, except Arg197 and Trp194, possibly via hydrophobic interactions (Ala104, Ile182, Val193, and Ile199). These results suggested that these six residues were key residues for erianin, especially Val193 and Arg197 with ∆Etotal of <−2.0 kcal/mol.

### 2.7. Confirmation of the Molecular Basis of Erianin against SrtA

**Figure 7:**
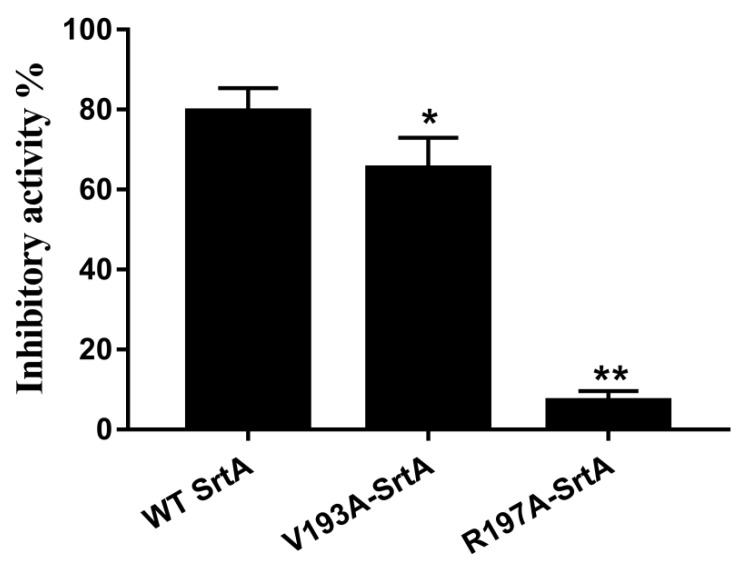
Inhibitory effects of erianin against WT-srtA and srtA mutants. WT-srtA and srtA mutants (V193A-srtA and R197A-srtA) were incubated with 64 μg/mL erianin, and the catalytic activity of recombinant srtA was determined as described in Figure 2. The error bars show the standard deviations (SD). * *p* < 0.05, ** *p* < 0.01 compared with WT-srtA.

To validate these theoretical results of the binding of erianin-srtA complex, we obtained two srtA-mutants (V193A-srtA and R197A-srtA) by point mutation. The total binding free energies (ΔG_bind_) of the interaction between WT-srtA, srtA mutants and erianin were calculated using an MM-GBSA approach. According to the calculation results (Table 1), the binding energy of WT-srtA was bigger than the mutants. That means that WT-srtA had the strongest ability to bind to erianin. The ΔG_bind_ and the number of binding sites between erianin and the two mutants were measured using fluorescence spectroscopy quenching, and the results were obtained by computational methods (Table 1). We found that these results showed consistent between the fluorescence spectroscopy quenching and MM-GBSA approach. To further confirm the simulation results, V193A-srtA and R197A-srtA were constructed for fluorescence resonance energy transfer (FRET) assay. The results of FRET assay (Figure 7) showed that the activity of erianin against V193A-srtA and R197A-srtA was significantly reduced compared with WT-srtA. These results showed that the information from the MD simulation on the srtA-erianin complex is reliable. Erianin inhibit the biological activity of srtA by binding to the active site region (residues of Ala92/Ala104/Ser116/Ala118/Ile182/Val193/Trp194/Arg197/Ile199).

### 2.8. Erianin Protected Mice from Fatal S. aureus Infection

**Figure 8:**
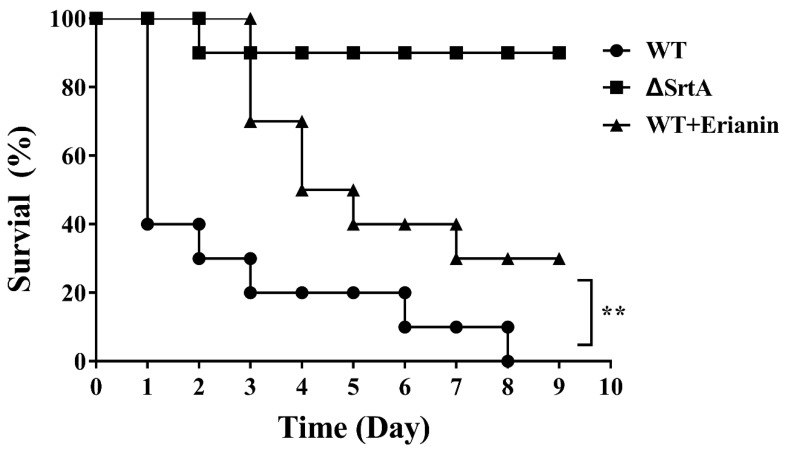
Effects of erianin on survival rates after 9-days monitoring of mice. Survival percentage of BALB/c mice (*N* = 10) after challenged with intravenous injection of 2 × 10^9^ CFU of *S. aureus* (WT) and ΔsrtA. Treatment with erianin (50 mg/kg, three times a day) was initiated 2 h after inoculation and again at 8-hour intervals. Survival statistics were calculated by Log-rank (Mantel–Cox) test. The statistical significance determined as follows: WT vs. ΔsrtA, ** *p* < 0.001.

The protective effects of erianin in vivo were determined by survival rate of mice infected with *S. aureus*. All mice inoculated with Newman D2C strain (WT group) died within 8 days. However, the mortality rate of mice infected with ΔsrtA strain was 10% (Figure 8). The *S. aureus*-inoculated mice were treated with erianin (50 mg/kg, three times per day) via subcutaneous injection. Nine days after infected, three mice still survive in the WT + erianin group (Figure 8). These results showed that erianin can increase the survival rate of mice infected with *S. aureus*. In addition, erianin cannot lysis rabbit red cells at the concentrations of 0–512 μg/mL (data not shown).

## 3. Discussion 

Virulence inhibitors are deemed new treatment strategies for bacterial infections, especially for multidrug-resistant bacterial [3,4,33]. Traditional antibiotics produce great pressures on bacterial growth during the process of administration, whereas virulence inhibitors produce low survival pressure on bacterial reproduction. In *S. aureus*, surface proteins and extracellular toxins are key virulence factors and play important roles in the adhesion, colonization, and destruction to host cells [1]. SrtA is a common and conserved transpeptidase found in many gram-positive bacteria [28]. SrtA anchors LEXTG-containing proteins to the gram-positive bacterial cell surface. In *S. aureus*, there are approximately 20 LEXTG-containing proteins, including fibronrctin-binding proteins (FnbpA and FnbpB), protein A (Spa), serine-aspartate repeat proteins (SdrC, ScdD and ScdE), collagen-binding protein (Cna) and clumping factors (ClfA and ClfB) [34]. These proteins play important roles in adhesion, biofilm formation, colonization, and evasion of the host innate immune defense in the pathogenicity of *S. aureus* [26]. SrtA inhibitors could affect the anchoring of many proteins to cell surface, a strategy which is better than targeting single surface protein for altering bacterial virulence [30]. Many natural and synthesized compounds capable of inhibiting the bioactivity of srtA in vivo and in vitro have been identified [35].

Our results showed that erianin (Figure 1) was a potential srtA inhibitor. In this study, erianin inhibited the activity of srtA and adhesion of *S. aureus* to fibrinogen at concentrations below the MIC without influencing the hemolysis ability of bacterial culture medium (data not shown). The IC_50_ of erianin against *S. aureus* srtA was 20.91 ± 2.31 μg/mL (65.7 ± 7.2 μM). We also evaluated the effect of erianin using a mouse infection model and found that it reduced their mortality rate. Erianin would be an assistance drug to against *S. aureus* infection. However, the exact mechanisms of erianin against *S. aureus* infection in vitro are not clear. TCM herbs exhibited the pharmacodynamics targeted different pathways with multi-components. Erianin is possible to act on the host by inhibiting the phlogistic pathways and improving the immunity, or interfere with several virulence factors of bacteria.

The molecular mechanism of erianin binding to srtA was also revealed using the molecular simulation methods. The results showed that erianin tightly binds to the residues in the active center of srtA. One previous study reported that the residues His120, Cys184 and Arg197, the β6/β7 loop and β7/β8 loop are essential for the catalysis by srtA [12]. The β6/β7 loop recognizes the substrate (LPXTG-containing proteins) of srtA [36,37]. Erianin binds to the β6/β7 loop via Van der Waals interaction (Ala104), hydrophobic interactions, and electrostatic interactions (Ala118). A comparison of free srtA and erianin-srtA complexes with respect to RMSF values of the residues indicated that erianin affected the flexibility of β6/β7 loop. Consequently, a combination of erianin and srtA would influence the recognition of the substrate, which is the first step of the process of protein anchoring by srtA. The mobility of β7/β8 loop in srtA provides a binding site for Lipid II [38,39]. The methoxy group of phenyl ring forms a hydrogen bond with Val193 (binding site of Lipid II in srtA) and places the β7/β8 loop into a closed state in the erianin-srtA complex. The thiol group of Cys184 bond to the carbonyl group of Thr (LPXTG-containing proteins) and form a thioester acyl enzyme intermediate [40]. Arg197 facilitates the cleavage of Thr and Gly through the ionization of Cys184 by forming a stabilized tetrahedral oxyanion transition state and providing the required activation energy [41]. In the docking model of erianin-srtA complex, the phenyl group of erianin was placed near Arg197, thus facilitating electrostatic interactions between them. Furthermore, cation–π interactions were observed between srtA and erianin. The phenyl rings of erianin formed a hydrophobic binding packet in the active site of srtA with the alky side chains of Ala92, Ala104, Asn114, Ser116, Ile182, Cys184, Trp194 and Ile199, which further stabilized the active center.

Oh et al. (2006) reported that (*Z*)-3-(2,5-dimethoxyphenyl)-2-(4-methoxyphenyl) acrylonitrile can inhibit the activity of srtA [42]. This compound has the same basic structural backbone of erianin. Erianin (IC_50_ = 20.91 ± 2.31 μg/mL, 65.7 ± 7.2 μM) has weaker activity in inhibiting srtA than (*Z*)-3-(2,5-dimethoxyphenyl)-2-(4-methoxyphenyl) acrylonitrile (IC_50_ = 2.7 µg/mL). Because of the structure of erianin, the stereohindrance due to trimethoxy groups make the ligand unavailable for srtA residues, and the hydroxyl enhances the hydrophilicity of erianin. The double bond and the cyano can increase the inhibitory activity [43].

These results suggested that erianin inhibited the anchoring of srtA by preventing the access and binding of the T-G peptide chain and Lipid II of the surface proteins to the bioactivity center. These results showed that erianin has the potential to treat *S. aureus* infections. Su et al. have reported that erianin basically had no cytotoxicity on human normal liver cell line L02 [21], although the toxicology of erianin will be research before it used as a therapeutic option against S. aureus infections in vivo. 

## 4. Materials and Methods

### 4.1. Expression and Purification of WT-SrtA, V193A-SrtA and R197A-SrtA

*S. aureus* strain Newman D2C (ATCC25904) was commercially obtained from the American Type Culture Collection (ATCC) and used in this study. The Newman D2C strain can produce srtA without hemolysins and coagulase. The ΔsrtA strain, which was constructed from Newman D2C, was graciously provided by Professor Deng, College of Veterinary Medicine, Jilin University [5]. The *strA* fragment was cloned from the *S. aureus* NewmanD2C genomic DNA by PCR. The *strA* fragment (the sequence express only residues 60–206) was cloned into the pGEX-6P-1 vector (GE Amersham), and made the recombinant plasmid pG-srtAΔN59 [44]. The mutations V193A and R197A were obtained from site-directed mutagenesis on the recombinant plasmid pG-srtAΔN59 using the QuickChange site-directed mutagenesis kit (Stratagene, La Jolla, CA, USA) according the manufacturer’s protocol. Electroporation was used to transfect the recombinant plasmid pG-srtAΔN59 and the mutant constructs into *Escherichia coli* strain BL21 (Invitrogen, Carlsbad, CA, USA). The transformed *Escherichia coli* were grown at 37 °C in LB broth with ampicillin (100 μg/mL). When bacteria showed an initial logarithmic growth phase initially (OD_600 nm_ = 0.6–0.8), isopropyl *β*-d-1-thiogalactopyr-anoside (IPTG, 1 mM) was added into the culture medium to induce the target protein. The bacteria were grown at 16 °C for 12–16 h. The recombinant proteins (WT-srtA, V193A-srtA and R197A-srtA) were released from the cells by sonication and dissolved in the reaction buffer [41]. These proteins solution were added into a GST-affinity column (2 mL glutathione Sepharose 4B; GE Amersham). These recombinant proteins were bounded to the GST column. The unbound proteins were washed using the reaction buffer. Proteins were concentrated by molecular size elution column and detected by SDS-PAGE. BCA protein assay kit (Pierce, Thermo Fisher Scientific, Shanghai, China) was used for determining the concentration of proteins. All expression vectors were confirmed via DNA sequencing. The mutagenic primer pairs employed to produce the three mutants are listed in Table 2.

### 4.2. Determination of Mutant and WT SrtA Activity

Erianin, which was purchased from Chengdu Herbpurify Co., Ltd., (Chengdu, China), was dissolved in Dimethyl sulfoxide (DMSO, Sigma, St. Louis, MO, USA). The solution was stored at 4 °C before use. The activities of erianin against WT-srtA, V193A-srtA and R197A-srtA were detected using the FRET assays. FRET assays were performed by disrupting a synthetic substrate peptide Dabcyl-QALPETGEE-Edans (GL Biochem, Shanghai, China) according to the protocols which have been published [7,14]. All reactions were performed in the black 96-well plate. Briefly, 300 μL of the reaction volume contained with reaction buffer, synthetic substrate peptide, recombinant proteins (WT-srtA, V193A-srtA and R197A-srtA), and different concentrations of erianin. The negative control contained all of the above components, except erianin. First, the mixture without synthetic substrate peptide was incubated at 37 °C for 30 min; then, the synthetic substrate peptides were added into the reaction system and incubated for 60 min at 37 °C. The sample fluorescence was analyzed at an emission wavelength of 495 nm and an excitation wavelength of 350 nm. We also checked the fluorescences of erianin co-culture with recombinant proteins, and synthetic substrate peptide. Each experiment was tested in triplicate to ensure reproducibility.

### 4.3. Susceptibility Testing and Growth Curve Assay

The minimal inhibitory concentrations (MICs) of erianin against *S. aureus* were measured using the broth microdilution method recommended by the Clinical and Laboratory Standards Institute. MIC was defined as the lowest concentrations of erianin that inhibited *S. aureus* growth. The negative control contained DMSO without erianin. For the growth curves, overnight cells cultures were grown in fresh brain-heart infusion (BHI) broth (Sigma) by diluted 1:100. When the OD_600 nm_ of the culture were reached 0.3, they were resuspended in a solution containing different concentrations of erianin (8, 16, 32, 64 and 128 μg/mL). DMSO was used as negative control group. The solutions were incubated with constant shaking (200 rpm) at 37 °C for different durations. The OD value was measured by UV-spectrophotometer (Agilent Technologies, Santa Clara, CA, USA) at 600 nm. 

### 4.4. Fibrinogen-Binding Assay

*S. aureus* cultures in the logarithmic phase were diluted to an initial OD_600 nm_ of 0.05 in BHI broth. Different concentrations of erianin were added to the *S. aureus* Newman D2C cultures. The Newman ΔsrtA strain was used as the positive control. The Newman D2C strain without erianin was as the wild type (WT) group. DMSO was used as negative control group. The mixtures were incubated at 37 °C with constant shaking (200 rpm) for 2 h. The bacteria were collected by centrifugation (5000× *g* for 5 min), and washed two times with sterile PBS, and the pellets were resuspended with PBS until use.

Bovine Fibrinogen (Sigma, 20 μg/mL) was seeded onto 96-well plates (Polystyrene Costar) and incubated at 4 °C overnight for coating. After washing, the plates were blocked with 5% bovine serum albumin (BSA, Sigma) at 37 °C. After 2 h, the plates were washed three times with sterile PBS. Then, the cell suspensions (100 μL/well) were added to the plates and incubated for 2 h at 37 °C. After removing the cells suspension, the adherent bacteria were fixed with formaldehyde (25%, *v*/*v*) for 30 min and stained with crystal violet (12.5 mg/mL) for 10 min. After washed with double distilled water and dried, 33% acetic acid was added to dissolve crystal violet. The absorbance of the sample was subsequently measured with a microplate reader (Thermo Scientific, Waltham, MA, USA) at 570 nm. The absorbance of the negative control group was used as the 100% adherence. The adherence rate of each sample was calculated by comparing to the negative control. 

### 4.5. Biofilm Formation Assay

*S. aureus* cultured overnight, and then were grown in fresh BHI broth by diluted 1:100 with erianin or DMSO at 37 °C with constant shaking (200 rpm). The Newman ΔsrtA strain was used as the positive control. The Newman D2C strain without erianin was as the wild type (WT) group. DMSO was used as negative control group. When the OD_600 nm_ of the culture are reached 0.6, 10 μL of the bacterial solution was added into 290 μL BHI broth containing 3% (*w*/*v*) sucrose. The mixture was placed in the 96-well flat-bottom polystyrene microliter plates, and incubated at 37 °C in anerobic box. After 18 h, we removed lightly the liquid mixture. And then, 100 μL of 10% formaldehyde solution was used to fix the biofilm at room temperature (RT) overnight. After removing the formaldehyde, crystal violet (12.5 mg/mL) was used to stain the biofilm at RT for 30 min. After washed with double distilled water and dried, 33% acetic acid was added to dissolve crystal violet. The absorbance of the sample was subsequently measured with a microplate reader (Thermo Scientific) at 490 nm.

### 4.6. Binding Affinity Determination of Erianin with Mutant and WT SrtA 

The binding constants (KA) of erianin with WT-srtA and mutant srtA were measured using the fluorescence-quenching method. A 280-nm excitation wavelength with a 5-nm bandpass and a 345-nm emission wavelength with a 10-nm bandpass were used for the measurements. The details of the measurements in this study have previously been described [45,46]. 

### 4.7. Molecular Docking and Molecular Dynamics

The binding mode between erianin and SrtA was investigated using molecular docking method with Autodock vina 1.1.2 [47]. The three-dimensional (3D) X-ray structure of srtA (PDB ID: 1T2P) used in this experiment was obtained from Protein Data Bank (http://www.rcsb.org/pdb/home/home.do). ChemBioDraw Ultra 12.0 and ChemBio3D Ultra 12.0 softwares were used to prepare the 3D structure of erianin. The docking input files were generated using Auto Dock Tools 1.5.6 package [48]. Ligand structures for docking were prepared by defining rotatable bonds and merging non-polar hydrogen atoms. The search grid for srtA was identified as center_x: −34.843, center_y: −17.649, and center_z: 7.103 with dimensions size_x: 12.75, size_y: 15 and size_z: 10.5. The value of exhaustiveness was set at 20 to increase the docking accuracy. Default parameters were used if parameter details were not mentioned in Vina docking. The docking result was revised using molecular dynamics (MD).

MD simulations of the selected docked positions were performed using the Amber 12 and AmberTools 13 programs [49,50]. The topologies and parameters of erianin were prepared by AnteChamber PYthon Parser interfacE (ACPYPE) [51]. Next, the force field of the ligand was prepared and labeled “leaprc.gaff” (generalized Amber force field), whereas the receptor was labeled “leaprc.ff12SB”. The details of MD simulation processes in this study have previously been described [52,53].

### 4.8. Animal Experiments

Mice (BALB/c) weighing 20 ± 2 g were commercially obtained from Chengdu Dossy Experimental Animals Co., Ltd., (Chengdu, China). All animal studies were carried out according to the experimental practices and standards of the animal ethics committee of Sichuan Agricultural University, and the experiment protocols were approved on 23 September 2016, and supervised by the animal care committee for project identification code 20160906.

The Newman D2C strain and Newman ΔsrtA strain were inoculated in BHI broth and incubated overnight at 37 °C. The cultures were diluted to 1:100 using fresh BHI broth and inoculated at 37 °C with constant shaking (200 rpm) for 3 h. The bacteria were collected by centrifugation (5000× *g* for 5 min at 4 °C) and washed two times with sterile PBS. Then, they were resuspended in fresh BHI broth to obtain staphylococcal suspension. In the survival studies, mice were infected with 100 μL of staphylococcal suspensions (2 × 10^9^ Colony-Forming Units–CFU) via tail vein injection [54]. Thirty mice were randomly divided into three groups in the survival studies: (1) mice infected with Newman D2C strain (WT group), (2) mice infected with Newman ΔsrtA strain (ΔsrtA group) and (3) mice infected with Newman D2C strain and treated with erianin (50 mg/kg, three times a day; WT + erianin group) for three days. Survival percentages of each group were recorded 9 days after infection. 

### 4.9. Statistical Analysis

The statistical significance of the percentage of Fg-binding was analyzed using the SPSS13.0 software (SPSS Inc., Chicago, IL, USA, 2005) with the unpaired two-tailed Student’s *t*-test. The significance of the survival studies was analyzed using Log-rank (Mantel-Cox). The differences were considered statistically significant when *p*-value was <0.05.

## Figures and Tables

**Table 1:** The binding free energy (kcal/mol) of WT-Erianin, V193A-Erianin and R197A-Erianin systems based on computational method and the values of the binding constants (K_A_) based on the fluorescence spectroscopy quenching.

Proteins	WT-SrtA	V193A-SrtA	R197A-SrtA
The binding energy	−24.1 ± 2.3	−23.8 ± 2.2	−18.9 ± 2.0
K_A_ (1 × 10^4^) L mol^−1^	44.5 ± 7.2	43.9 ± 6.7	39.6 ± 4.8

**Table 2:** Oligonucleotide primers used in this study.

Primer Name	Oligonucleotide (5-3) ^a^
PsrtA59F	GCGGGATCCCCGGAATTCCAAGCTAAACCTCAAATTCC
PsrtA59R	CCGCTCGAGTTATTTGACTTCTGTAGCTACAA
V193A-srtA-F	TGAAAAGACAGGCGCTTGGGAAAAAC
V193A-srtA-R	TTCCCAGCGCCTGTCTTTTCATTGTAATCAT
R197A-srtA-F	GACAGGCGTTTGGGAAAAAGCGAAAATCTTTGTAGCTACAG
R197A-srtA-R	CTGTAGCTACAAAGATTTTCGCTTTTTCCCAAACGCCTGTC

^a^ Restriction endonuclease recognition sites or mutated codons are underlined.
